# Quantitative ADC: An Additional Tool in the Evaluation of Prostate Cancer?

**DOI:** 10.3390/jpm13091378

**Published:** 2023-09-15

**Authors:** Nicola Maria Lucarelli, Ilaria Villanova, Nicola Maggialetti, Sara Greco, Francesca Tarantino, Roberto Russo, Senia Maria Rosaria Trabucco, Amato Antonio Stabile Ianora, Arnaldo Scardapane

**Affiliations:** 1 Interdisciplinary Department of Medicine, Section of Radiology and Radiation Oncology, University of Bari “Aldo Moro”, 70124 Bari, Italy; lucarelli.nico@gmail.com (N.M.L.); ila.vill@libero.it (I.V.); n.maggialetti@gmail.com (N.M.); saragrecosa@gmail.com (S.G.); dottor.roberto.russo@gmail.com (R.R.); amatoantonio.stabileianora@uniba.it (A.A.S.I.); arnaldo.scardapane@gmail.com (A.S.); 2 Section of Molecular Pathology, Department of Emergency and Organ Transplantation (DETO), University of Bari “Aldo Moro”, 70124 Bari, Italy; senia.traucco@policlinico.ba.it

**Keywords:** prostate cancer, magnetic resonance imaging (MRI), quantitative apparent diffusion coefficient (ADC), diffusion-weighted imaging (DWI), PIRADS, ISUP

## Abstract

Prostate cancer is one of the most common tumors among the male population. Magnetic resonance imaging (MRI), standardized by the PI-RADS version 2.1 scoring system, has a fundamental role in detecting prostate cancer and evaluating its aggressiveness. Diffusion-weighted imaging sequences and apparent diffusion coefficient values, in particular, are considered fundamental for the detection and characterization of lesions. In 2016 the International Society of Urological Pathology introduced a new anatomopathological 5-grade scoring system for prostate cancer. The aim of this study is to evaluate the correlation between quantitative apparent diffusion coefficient values (ADC) derived from diffusion-weighted imaging (DWI) sequences and the International Society of Urological Pathology (ISUP) and PI-RADS groups. Our retrospective study included 143 patients with 154 suspicious lesions, observed on prostate magnetic resonance imaging and compared with the histological results of the biopsy. We observed that ADC values can aid in discriminating between not clinically significant (ISUP 1) and clinically significant (ISUP 2-5) prostate cancers. In fact, ADC values were lower in ISUP 5 lesions than in negative lesions. We also found a correlation between ADC values and PI-RADS groups; we noted lower ADC values in the PI-RADS 5 and PI-RADS 4 groups than in the PI-RADS 3 group. In conclusion, quantitative apparent diffusion coefficient values can be useful to assess the aggressiveness of prostate cancer.

## 1. Introduction

Prostate cancer is one of the most common malignancies in men worldwide [1] and represents over 20% of all cancers diagnosed from 50 years of age [2].

Despite the high incidence, survival is currently attested to 91.4% at 5 years after diagnosis. The increase in survival and the development of minimally invasive procedures have caused a rising interest in the detection of prostate cancer.

A pretreatment estimation of cancer aggressiveness allows for timely diagnosis for patients with highly aggressive tumors that need prompt therapy and avoids overtreatment of indolent cancers [3]. The main problem for the early detection of prostate cancers is that most of them are usually asymptomatic in the earliest stages. In the advanced stages, the increase in the size of the neoplasm can cause compression of the prostatic urethra and lower urinary tract symptoms (LUTS).

However, these clinical manifestations are not specific to prostate cancer, and they can also be caused by benign prostatic pathologies such as benign prostatic hypertrophy (BPH) [4,5].

Digital rectal examination (RE), prostate-specific antigen (PSA) testing, imaging such as transrectal ultrasound (TRUS) and magnetic resonance imaging (MRI), and prostate biopsy contribute to the prostate cancer diagnosis.

RE should be the first diagnostic approach to patients with symptoms suspected of prostate pathology. A palpatory suspicion at the RE, associated with a PSA > 2 ng/mL, has a positive predictive value between 5 and 30% [2,3,4,5,6]. However, due to its low sensibility and specificity, RE cannot be used individually as the only diagnostic method.

PSA testing remains the landmark for prostate cancer screening.

PSA is a glycoprotein produced by the epithelial cells of the prostate gland and secreted in the semen. The transformation of the normal prostate histoarchitecture, as happens in the case of benign pathology (prostatic hypertrophy or prostatitis) and also in the case of prostate cancer, causes an increase in blood levels of the PSA which must therefore be considered a marker of prostate disease [2].

The PSA reference value cut-off is 4 ng/mL, so a patient with PSA levels above 4 ng/mL needs supplemental tests, since benign prostatic diseases can also cause the rise of PSA values. PSA levels between 4 ng/mL and 10 ng/mL suggest that the possibility of having prostate cancer is about 25%. If the PSA level is higher than 10 ng/mL, the risk of prostate cancer is over 50% [1].

Due to the poor diagnostic specificity of PSA test, some of its variants, called PSA derivatives (free/total PSA ratio, PSA density, and PSA velocity), have been devised to optimize its use in the selection of clinically significant tumors.

A lower free/total PSA ratio is associated with a higher risk of prostate cancer.

High PSA density (PSA level divided by prostate volume expressed in nanograms per milliliter per cubic centimeter, ng/mL/cc) values are suspicious for cancer. In fact, values lower than 0.1 ng/mL/cc suggest a BPH, while values higher than 0.15 ng/mL/cc are suggestive of carcinoma. According to the current literature, this parameter is an indicator of reduced risk of aggressive disease in cases of negative or doubtful MRIs: a PSA density value lower than 0.15 ng/mL/cc would identify men with lower probabilities of having clinically significant prostate cancer who could therefore avoid biopsy [7,8,9].

PSA velocity expresses the rate of increase of PSA levels over time; an annual increase of more than 2 ng/mL is considered to be suspicious for neoplasia [7].

Imaging methods, such as TRUS and MRI, are useful in the evaluation of the prostatic gland.

TRUS is performed with the bladder partially distended, after the RE. The suspect areas are hypoechoic, mostly found in the peripheral area, which is not very echogenic with clear or sometimes blurred margins, is often not uniform with rare microcalcifications, and is unevenly vascularized [10]. However, although most prostate cancers present with hypoechoic lesions, some cancers can be hyperechoic or isoechoic [2].

TRUS has limited sensitivity and specificity for the detection of prostate cancer, with approximately 30% of malignancies being unrecognizable and only 21–56% of detected hypoechoic nodules being carcinomas. The suspicion of malignancy of the lesion increases in cases of large size, RE hardness, and an increase in PSA.

Therefore, integrating functional and morphological information, MRI is the most accurate and used instrumental noninvasive method for the assessment of suspicious cancerous lesions [11]. It is also useful to follow-up with men on active surveillance or with prior negative biopsies [12].

The examination of the prostate in MRI is standardized by the PI-RADS version 2.1 scoring system.

A PI-RADS score of 1 or 2 is highly probable to not be cancer. PI-RADS grade 3 is doubtful; indeed, lesions classified as PI-RADS 3 commonly reveal benign histology on biopsy, but low-grade prostate cancer is possible and intermediate- or high-grade cancers cannot be entirely excluded. A PI-RADS score of 4 or 5 has a high probability to be a clinically significant disease. Histological confirmation with a biopsy is recommended for lesions of PI-RADS ≥ 3. [13,14,15,16].

This classification system foresees the use of different sequences (T2-weighted sequences, diffusion-weighted imaging (DWI) sequences, and pre-contrast and post-contrast T1-weighted sequences) which together increase the diagnostic accuracy of the MRI in recognizing prostate cancer [11].

Prostate cancer is frequently located in the peripheral zone, in which DWI sequences and apparent diffusion coefficient (ADC) maps, obtained by the analysis of acquired data, are considered as the dominant tools for the detection and characterization of lesions.

DWI and the corresponding ADC maps, in particular, can be used to subclassify tumors according to aggressiveness.

Two gradient pulses (diffusion gradient pulses) are added before and after a 180° pulse to make a diffusion-sensitive pulse sequence. In the case of stationary spins, the dephasing of the spin due to the first diffusion gradient is followed by a perfect rephasing by the second gradient. In the case of a spin in motion, the power factor correction will be incomplete, with the consequent loss of signal inside the voxel [17].

DWI is a powerful clinical tool that provides functional information about tissues at the cellular level [16].

The DWI sequence exploits the principle of “Brownian” motions, according to which the diffusive properties of tissues are directly related to the motion of interstitial free water and the degree of permeability. It is also sensitive to the restricted diffusion that occurs in tumors due to increased cellularity [18].

In prostate cancer, normal architecture is altered, in which the large interstitial spaces and the glandular lumens are replaced by nests of tumor cells and fibrous stroma with a consequent reduction in the movements of free water.

In particular, tumor tissue tends to have lower diffusivity than normal tissue due to its high cellularity [19].

So, the high-intensity signal zone on DWI is suggestive for clinically significant cancer. However, there is considerable overlap between BPH and prostatic cancer, as a different DWI signal intensity could be shown in the normal architecture of the gland.

Transition and peripheral zones have different structures, and this results in different DWI signal intensity, depending on the relative amount of glandular or stromal tissue.

The b-value determines the sensitivity of the DWI sequence in identifying the zones of increased diffusivity [20].

The b-value measures the degree of diffusion weighting applied, indicating the amplitude (G), the time of applied gradients (δ), and the duration between the paired gradients (Δ), and is calculated as b = γ^2^ G^2^ δ^2^ (Δ − δ/3) [21].

A larger b-value is achieved by increasing the gradient amplitude and duration and by widening the interval between paired gradient pulses.

To increase the ADC map’s accuracy, DWI should include a high b-value.

The ADC map is a model that expresses the signal decay with an increased b-value. Its accuracy is widely accepted, with a sensitivity and specificity of 82.6% and 91.3%, respectively, and a positive and negative predictive value of 100% [22].

So, ADC may be helpful in assessing the aggressiveness of prostate cancer lesions and clinical risk, and it has shown an increasing accuracy in addition to the DWI analysis in the detection and localization of prostate cancer [11].

According to the ESUR guidelines, it is advisable to use at least two b-values to obtain an ADC map, with the lower at 50–100 s/mm^2^ and the higher ranging from 800–1000 to 2000 s/mm^2^ [23].

As the b-value increases, the signal-to-noise ratio (SNR) decreases, so the optimum high b-value may be dependent on the magnetic field strength, software, and manufacturer.

Nowadays, there is no widely accepted “high b-value”; the b-values used are often more than 1000 s/mm^2^, with a maximum b-value ranging from 2000 to 3000 s/mm^2^ [24].

DWI must always be associated with the evaluation of the ADC maps for the “T2 shine through effect”. As the factor b increases, the T2 weighting of the image progressively decreases and the diffusion weighting progressively increases.

Currently, the indication for the execution of the prostate biopsy and the histopathological examination are formulated on the basis of the clinical and/or laboratory suspicion and/or a positive result of the MRI [25].

US-guided prostate biopsy is currently the diagnostic gold standard and is performed by a trans-rectal or trans-perineal approach. In patients with positive MRI, prostate biopsies can be performed with three different approaches: cognitive, MRI-guided (fusion biopsy), or MRI-guided (in-bore).

The use of cognitive biopsies performed under ultrasound guidance on the basis of MR images (without digital image fusion) is now outdated, as evidenced by a series of studies comparing cognitive vs. fusion approaches [26]. It is advisable to carry out a minimum of three samples for each suspicious area [27].

Furthermore, in patients undergoing targeted prostate biopsies on suspicious MRI, it is recommended to also include systematic biopsies on other regions of the prostate gland.

The fusion technique requires MR images. Real-time TRUS-MRI fusion is achieved to create a three-dimensional reconstruction of the prostate, which is targeted and tracked to the biopsy site [28].

An advantage of fusion biopsy is the possibility of targeting lesions that are not visualized only with US and targeting areas that are obscured by large calcifications.

Furthermore, another advantage is the opportunity to record the biopsy site, which can be useful for the follow-up of patients undergoing active surveillance [29].

A potential limitation of fusion biopsy may be in the quality of the fusion between MRI and TRUS images. Inaccurate segmentation of MRI or TRUS images can lead to misregistration.

Advances in imaging technology as well as improvements in prostate biopsy techniques may help increase the development of focal therapy, offering an alternative to the treatment of the whole gland [30].

Various focal approaches can be used, such as high-intensity focused ultrasound, cryotherapy, hyperthermia ablation, and transurethral ultrasound ablation.

All focal modalities are associated with fewer side effects regarding incontinence and erectile dysfunction, although population-based studies that examined the utilization of focal therapies for prostate cancer are scarce [31].

The histological grade of prostate cancer is expressed by the Gleason score.

The Gleason score (from 2 to 10) is assigned based on the most represented (primary grade) and second most represented (secondary grade) structural features in the neoplasm. When there is no secondary grade, one must double the primary grade to obtain the Gleason score. Gleason grade patterns are assigned from 1 to 5, based on the characteristics of the gland.

In 2016, the International Society of Urological Pathology (ISUP) introduced a pathologic 5-grade scoring system for prostate cancer in order to facilitate communication, simplifying the number of grading categories. These five grade groups were established according to the Gleason score.

ISUP grade 1 includes Gleason scores ≤ 6; ISUP grades 2 and 3 correspond to Gleason score 3 + 4 = 7 and Gleason score 4 + 3 = 7, respectively; ISUP grade 4 includes Gleason scores 4 + 4 = 8, 3 + 5 = 8, and 5 + 3 = 8; and grade 5 includes the Gleason scores 9–10 [32] (Table 1).

The aim of this study is to examine the potential correlation between ADCs derived from DWI MRI, and the ISUP and PI-RADS groups. ADC map has shown an overall high correlation with Gleason and ISUP scores [16,33].

Even though there have been several previous studies that have evaluated the role of DWI for the assessment of prostate cancer, most of these have focused on the correlation between ADC values and Gleason scores. Therefore, our aim is to demonstrate that ADC values correlate with ISUP and can help discriminate between negative lesions (N), not clinically significant prostate cancers (ISUP group 1), and clinically significant (ISUP groups 2–5) prostate cancers.

## 2. Materials and Methods

### 2.1. Study Sample

In this retrospective study, we evaluated a population of 143 patients with 154 suspicious lesions who underwent prostate MRI at the Policlinico of Bari between May 2019 and September 2022.

The examination was requested by the urologist specialist, or sometimes by the general practitioner, for patients with an increasing serum PSA value over time or with a single high value during a first blood chemistry test—in addition to this, the urological examination often documented a focal area of increased consistency on RE and/or a suspicious nodule on TRUS examination—or the patient was being followed-up with for a known nodule described in a previous MRI of dubious diagnostic significance, of small size, or that had already undergone biopsy with negative results of a precancerous condition. More rarely, the patient voluntarily presented himself to our attention, even in the absence of elevated serum PSA values or a previous urological visit, reporting because he wanted to undergo the investigation for screening and/or due to a family history positive for prostate cancer.

The patient was usually asymptomatic or complained of general symptoms of dysuria, nocturia, or stranguria.

We included patients with one or more suspicious lesions with PI-RADS scores from 3 to 5.

Instead, we excluded all patients with prostate glands free from lesions or those with findings of benignity compatible with PI-RADS categories 1 and 2.

Moreover, patients in whom the examination had been prematurely interrupted due to their explicit request due to a claustrophobic crisis or for other reasons were also excluded, as well as those in whom the contrast medium was not administered for an absence of blood tests proving adequate renal function or a lack of consent to the injection.

A previous surgical history positive for radical prostatectomy, transurethral resection of the prostate (TURP), and other prostate surgery represented an exclusion criterion since these conditions are not compatible with the assignment of the PI-RADS score. Patients who underwent radical prostatectomy were furthermore excluded because there could not be the certainty that any clinically significant prostate cancer detected by the anatomopathologist corresponded to the lesion reported by the radiologist.

A further exclusion criterion was identified in a previous prostate biopsy, in order to obtain a population of naïve patients, in which neither the radiologist nor the other specialists of the multidisciplinary team could be conditioned by previous imaging and biopsy findings.

**Figure 1:**
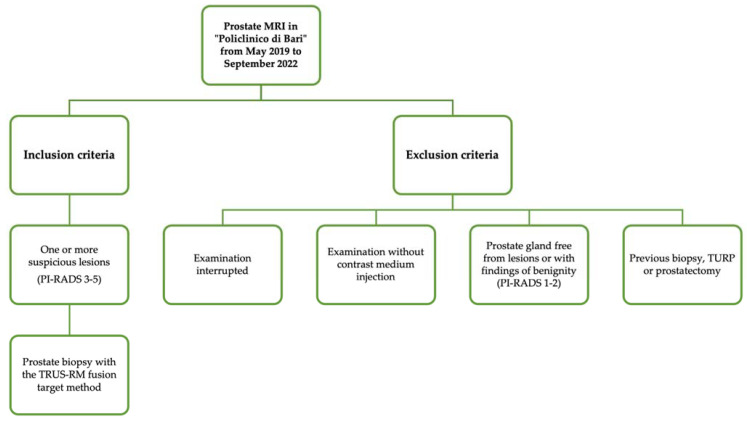
Flowchart of inclusion and exclusion criteria.

We included patients with PI-RADS scores ≥ 3 who underwent prostate biopsy with the TRUS-MR fusion target method, based on the lesions reported during the MRI examination (Figure 1).

Finally, the histological results of the biopsy were compared with the lesion observed on MRI.

As reported in the PI-RADS guidelines in version 2.1, tumors associated with a ISUP score from 2 to 5 (Gleason score greater than or equal to 7) were considered clinically significant; any lesions corresponding ISUP 1 (Gleason score 3 + 3) were therefore considered not clinically significant.

### 2.2. MRI Protocol and Reporting

The MRI studies were performed using a 1.5T scanner (Philips Achieva Nova Dual, Philips Medical Systems, Best, The Netherlands) with a phased array surface coil. A routine protocol included triplanar T2-weighted imaging using the parameters: repetition time (ms)/echo time (ms), 3000–5000/110; section thickness, 3 mm; field of view, 160 × 160; matrix, 240 × 168. DWI was acquired with the following b values: 0 s/mm^2^, 700 s/mm^2^, 1000 s/mm^2^, and 1400 s/mm^2^. ADC map was constructed based on the mono-exponential model. DCE imaging were performed using the parameters: repetition time (ms)/echo time (ms), 3.4–3.5/1.4; section thickness, 6 mm; field of view, 200 × 200; matrix, 240 × 168. Extracellular gadolinium-based contrast media was injected at a dose of 0.2 cc/kg and a rate of 2 cc/s.

All MRIs were also archived using our institutional PACS (Picture Archiving and Communication System) Carestream Health, Rochester, NY, USA.

### 2.3. Imaging Assessment

Using a freehand ROI, we measured mean ADC values (in square millimeters per second × 10^−3^) in each lesion. The ROI includes the largest area possible of the lesion on the axial plane on a single image without including the lesion margins to avoid contamination by the surrounding tissues.

ADC values were calculated independently by 2 radiologists (A.S. and N.M., with 23 and 12 years of experience, respectively); disagreements were resolved by open discussion and consensus from all the authors.

ADC values in each lesion were compared with the ISUP group after TRUS-RM fusion target biopsy.

### 2.4. Statistical Analysis

All statistical analyses were performed using SPSS software (version 26.0 SPSS Inc., Armonk, NY, USA).

The continuous variables are expressed as mean ± standard deviation (SD) and the categorical variables are given as percentages.

Spearman’s rank correlation was used to evaluate the relationship between ADC values and ISUP scores and between ADC values and PIRADS categories. *p*-value < 0.05 was considered statistically significant.

Diagnostic accuracy of the ADC value was assessed by receiver operating characteristic (ROC) analysis.

## 3. Results

Our cohort was composed of 143 patients with one or more prostate lesions, for a total of 154 lesions with PI-RADS scores ≥ 3, considering both peripheral (112/154 lesions; 73%) and transition zone (42/154 lesions; 27%) lesions.

Radiologists evaluated suspicious lesions according to the PI-RADS version 2.1 criteria.

**Figure 2:**
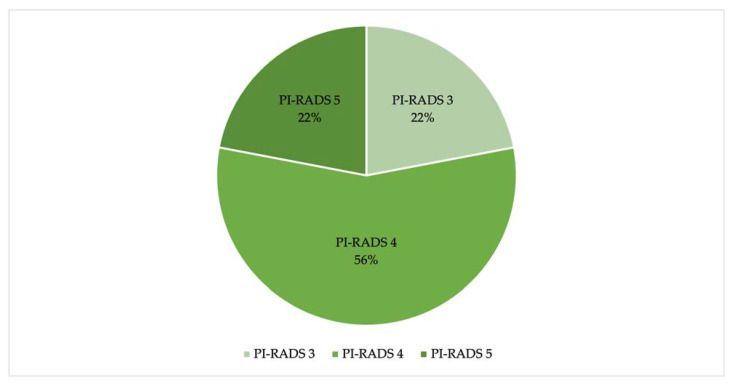
Percentage of lesions for each PI-RADS group.

PI-RADS scores of 1 and 2 were excluded. Instead, the radiologists assigned PI-RADS 3 to 22% of the lesions (34/154 lesions), PI-RADS 4 to 56% of the lesions (86/154 lesions), and PI-RADS 5 to 22% of the lesions (34/154 lesions) (Figure 2).

The anatomopathological analysis of the samples taken by target biopsy revealed the presence of benign lesions (inflammation, atrophy, and stromal hyperplasia) (N) (65/154 lesions; 42%) and malignant lesions corresponding to ISUP scores between 1 and 5.

**Figure 3:**
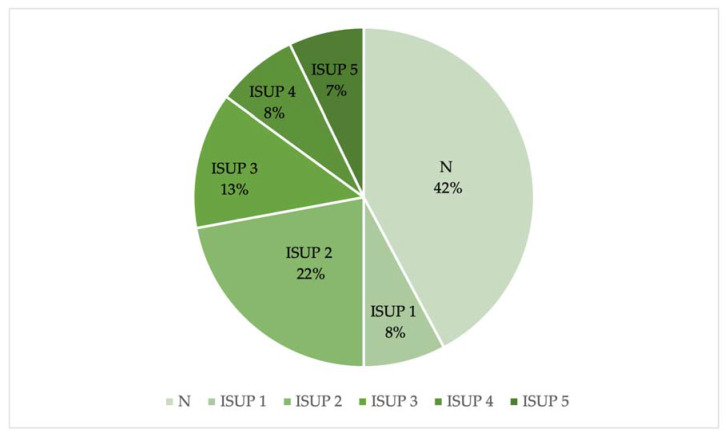
Percentage of benign lesions (N) and malignant lesions (ISUP 1–5) based on the anatomopathological results.

ISUP 1 scores were considered not clinically significant (12/154 lesions; 8%), while scores from ISUP 2 to ISUP 5 were considered clinically significant (77/154 lesions; 50%) (Figure 3).

**Figure 4:**
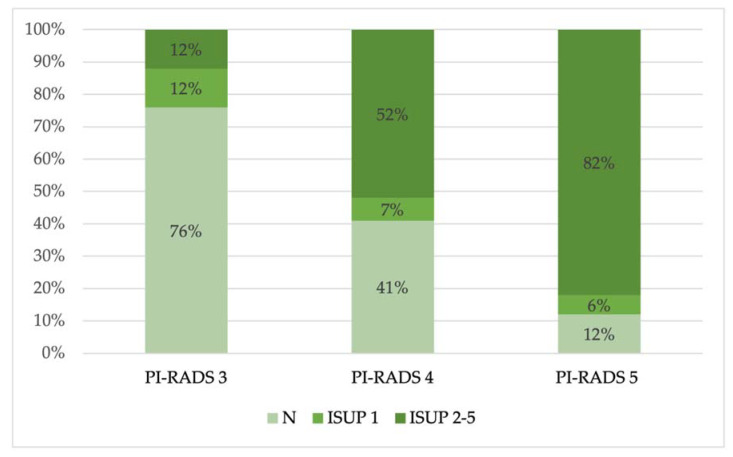
Percentage of benign (N) and malignant lesions (ISUP 1–5) in each PI-RADS group.

Then, we observed the prevalence of benign and malignant lesions (divided in ISUP 1 and ISUP 2–5) in each PI-RADS group (Figure 4).

However, our study focuses on evaluating the correlations between ADC values and ISUP groups and between ADC values and PI-RADS groups.

We observed that ADC values can aid in discriminating between negative lesions (N), not clinically significant prostate cancers (ISUP group 1), and clinically significant (ISUP groups 2–5) prostate cancers.

In fact, ADC values were lower in ISUP 5 lesions (mean ADC values 0.72 mm^2^/s × 10^−3^ ± 0.12) than in negative lesions (1.00 mm^2^/s × 10^−3^ ± 0.13).

The mean ADC values were 1.00 mm^2^/s × 10^−3^ in negative lesions, 0.79 mm^2^/s × 10^−3^ in ISUP 1 lesions, and 0.74 mm^2^/s × 10^−3^ in ISUP 2–5 lesions.

**Figure 5:**
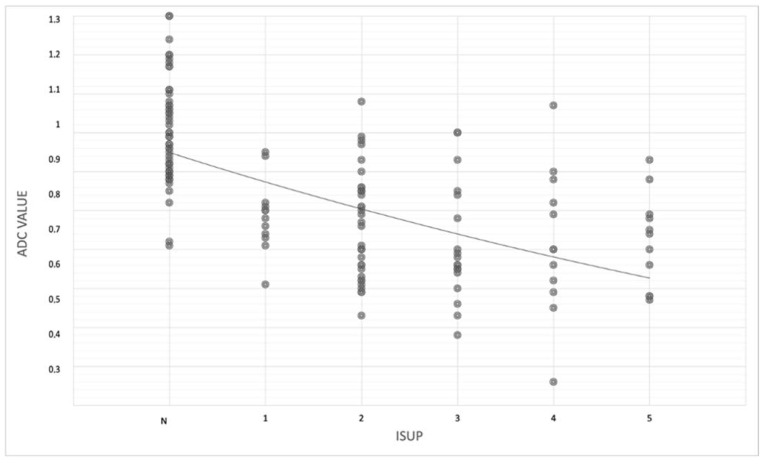
Scatterplot showing correlation between mean ADC values and benign and ISUP (from 1 to 5) lesions in both peripheral and transition zones.

In particular, mean ADC values decrease from ISUP 2 to ISUP 5 (0.76 mm^2^/s × 10^−3^ in ISUP 2, 0.73 mm^2^/s × 10^−3^ in ISUP 3, 0.72 mm^2^/s × 10^−3^ in ISUP 4, and 0.72 mm^2^/s × 10^−3^ in ISUP 5) (rho − 0.65; *p*-value < 0.05) (Table 2 and Figure 5).

**Figure 6:**
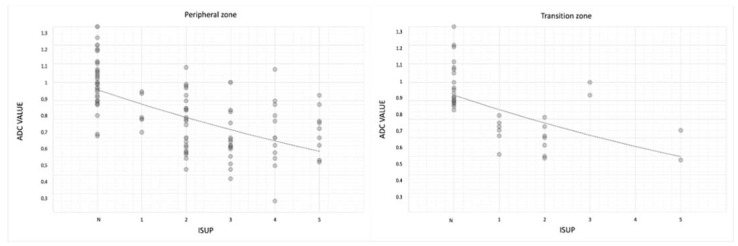
Scatterplot showing relationship between mean ADC values and benign and ISUP (from 1 to 5) lesions divided into peripheral and transition zones.

Mean ADC values were lower in ISUP 5 than in negative lesions both in the transition and peripheral zones (0.73 ± 0.14 in transition zone; 0.74 ± 0.15 in peripheral zone) (Table 3 and Figure 6).

For the transition zone, (rho −0.67; *p*-value < 0.05); and for the peripheral zone, (rho −0.64; *p*-value < 0.05) (Table 4).

Then, we observed the correlation between mean ADC values and the PI-RADS group, and we noted lower ADC values in the PI-RADS 5 group than in the PI-RADS 3 group.

**Figure 7:**
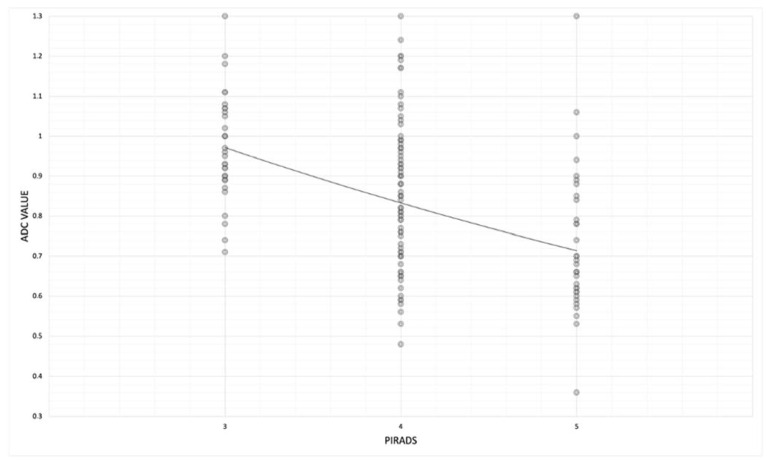
Scatterplot showing relationship between mean ADC values in each PI-RADS group (from 3 to 5) in both peripheral and transition zones.

In fact, in the PI-RADS 3 group, mean ADC values were 0.97 ± 0.13 and decrease in PI-RADS 4 and 5, where mean ADC values were, respectively, 0.86 ± 0.18 and 0.73 ± 0.18. (rho − 0.47; *p*-value < 0.05) (Table 5 and Figure 7).

**Figure 8:**
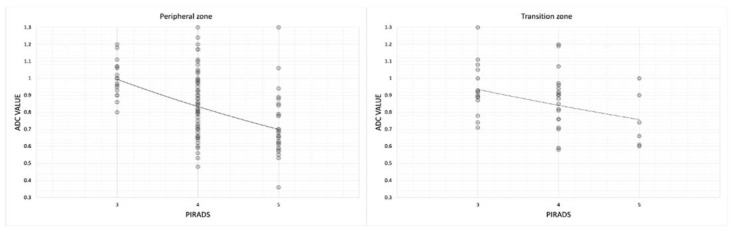
Scatterplot showing relationship between mean ADC values in each PI-RADS group (from 3 to 5) divided in peripheral and transition zones.

**Figure 9:**
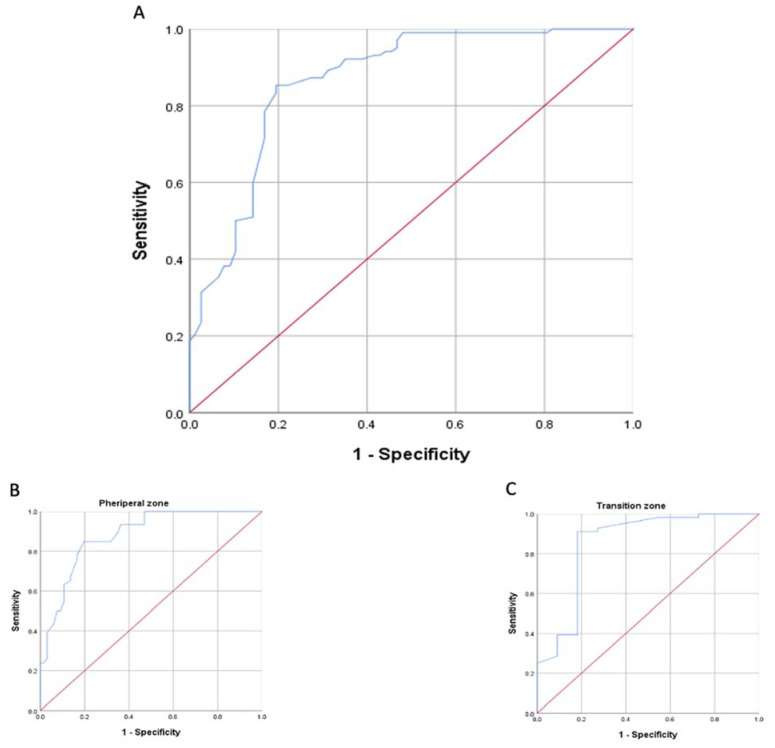
ROC curve of the ADC values (**A**) stratified by tumor zone of origin (**B**,**C**). The area under the ROC curve (AUC) suggests that ADC value is a reasonable predictor for differentiating diagnoses of clinically significant prostate cancer.

Then, we noted that in both the transition and peripheral zones, mean ADC values were lower in PI-RADS 5 (0.75 ± 0.16 in transition zone and 0.72 ± 0.18 in peripheral zone) than in PI-RADS 3 (0.94 ± 0.15 in transition zone and 1.00 ± 0.11) (Table 6, Figure 8, Table 7, Figure 9 and Table 8).

## 4. Discussion

Diagnostic imaging has achieved high accuracy in detecting tumors and defining their characteristics and aggressiveness [34].

MRI is well-known to provide better soft tissue contrast than conventional morphological imaging techniques such as ultrasound or computed tomography (CT). For this reason, MRI may ensure a proper definition of the anatomy of the pelvic organs and of the prostate and prostate bed boundaries [35].

This evidence is confirmed by the current role of multiparametric prostate MRI, which is the most used instrumental method for the diagnosis of prostate cancer, combining morphological and functional data [36,37].

The evaluation of suspected prostate cancer in naïve prostate glands is standardized by the PI-RADS version 2.1 criteria, a structured reporting scheme for MRI published and developed in 2019 by the American College of Radiology (ACR), European Society of Urogenital Radiology (ESUR), and AdMeTech Foundation [38].

PI-RADS version 2.1 foresees the use of different sequences such as T2-weighted sequences, DWI/ADC, and pre-contrast and post-contrast T1-weighted sequences, which together increase the detective capacity of the magnetic resonance in recognizing prostate cancer.

Multiparametric prostate MRI, combined with a growing interpreter experience, has improved the detection of benign diseases and clinically significant cancer in order to optimize clinical pathways.

According to the literature, we noted that in our cohort, most of the lesions arise in the peripheral zone; in fact, 73% of the lesions were in the peripheral zone, while 27% were in the transitional zone.

This allows us to make the most of the DWI sequences with the related ADC maps, since the DWI is the key sequence in the peripheral zone.

Based on the PI-RADS criteria, radiologists assigned PI-RADS 3 to 34 lesions (22%), PI-RADS 4 to 86 lesions (56%), and PI-RADS 5 to 34 lesions (22%) (Figure 2).

Then, we compared the PI-RADS grades with the anatomopathological results, and we noted that 50% of the lesions were benign (N) or not clinically significative (ISUP 1) (77/154 lesions), according to previous publications, while the remaining 50% consisted of clinically significant lesions (ISUP 2 to 5) (Figure 3); in particular, in the PI-RADS 4 and PI-RADS 5 groups, most lesions were clinically significant (52% and 82%, respectively), while in the PI-RADS 3 group, most lesions were benign (N) (76%) (Figure 4).

According to the current literature, this confirms that PI-RADS grades are a useful tool to guide us about the likelihood of clinically significant cancers.

In our study, we focused on the utility of quantitative ADC; indeed, unlike DWI sequences, ADC maps provide values that are quantitative measures, so improving the utilization of ADC as a quantitative imaging marker would allow us to meliorate the detection of prostate cancer by reducing inter-radiologist subjectivity and therefore decreasing overdiagnosis; this can permit us to avoid unnecessary biopsies and to reduce overtreatment of indolent prostate cancer. Considering that ADC maps are reproducible, these could be used, potentially, as a noninvasive method to follow-up with patients with low clinical risk and negative previous biopsies, instead of repeating biopsy [12].

Indeed, there is an increasing concern in the employment of MRI for men on active surveillance and on follow-up, as several studies show [39,40].

ADC mean values should be potentially added to other MRI sequences and other clinical sources such as Gleason or ISUP scores, PSA velocity, and density helping to recognize the lesion grade and select patients best-suited to undergo active surveillance [17,41,42,43].

We observed how ADC values correlate with ISUP groups and PI-RADS groups in both peripheral and transition zones.

The mean ADC values were 1.00 mm^2^/s × 10^−3^ in negative lesions, 0.79 mm^2^/s × 10^−3^ in ISUP 1 lesions, and 0.74 mm^2^/s × 10^−3^ in ISUP 2–5 lesions, and, in particular, mean ADC values decrease from ISUP 2 to ISUP 5 (Table 2 and Figure 5) (*p* < 0.05).

Furthermore, both in peripheral and in transition zones, the ADC value correlates with the ISUP grade; indeed, mean ADC values were lower in ISUP 5 than in negative lesions both in transition and peripheral zones (0.73 mm^2^/s × 10^−3^ ± 0.14 in transition zone; 0.74 mm^2^/s × 10^−3^ ± 0.15 in peripheral zone) (Table 3 and Figure 6) (*p* < 0.05).

Therefore, the ADC values correlate well with the histology of the lesion both in the peripheral zone and in the transition zone, with statistically significant results (*p* < 0.05) (Table 4).

**Figure 10:**
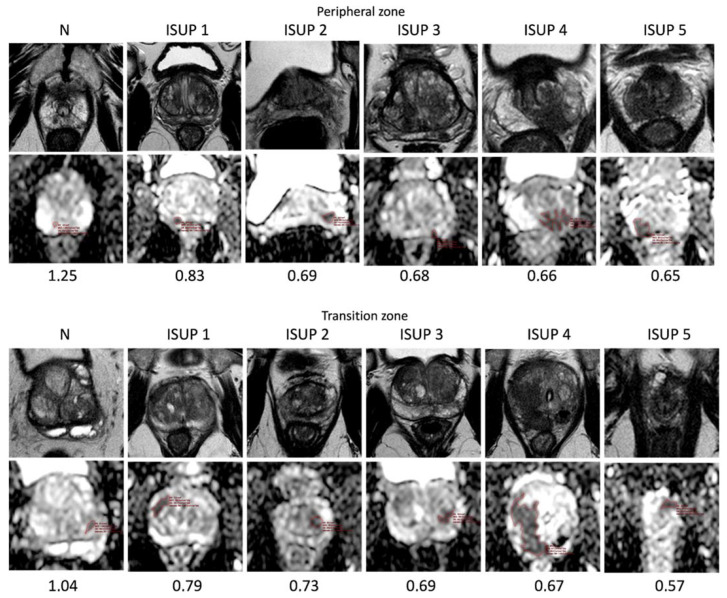
Examples of ADC values (mm^2^/s × 10^−3^) in different zones of the prostate, classified according to different ISUP groups.

This can be explained by the structural changes of gland stroma, becoming more fibrous, and the increase in tumor cell density that leads to a more restricted motion of water molecules within more aggressive cancers. Moreover, a significant difference was also observed between the mean ADC values of low-, intermediate-, and high-clinical-risk tumors (Figure 10).

For both peripheral and transition zones, the top row shows the axial T2 images, the middle row displays the ADC maps with the freehand ROI, and the last row presents the ADC values.

We observed the correlation between the mean ADC values and the PI-RADS groups, and we noted lower ADC values in the PI-RADS 5 group (0.73 mm^2^/s × 10^−3^ ± 0.18) than in the PI-RADS 3 group (0.97 mm^2^/s × 10^−3^ ± 0.13) (Table 5 and Figure 7).

Then, we noted that both in the transition and peripheral zones, the mean ADC values were lower in PI-RADS 5 (0.75 mm^2^/s × 10^−3^ ± 0.16 in transition zone and 0.72 mm^2^/s × 10^–3^ ± 0.18 in peripheral zone) than in PI-RADS 3 (0.94 mm^2^/s × 10^−3^ ± 0.15 in transition zone and 1.00 mm^2^/s × 10^−3^ ± 0.11) (Table 6 and Figure 8) (in transition zone, *p* = 0.04; in peripheral zone, *p* < 0.05) (Table 7).

This emphasizes the central role of the DWI and the ADC values in the peripheral zone, while in the transition zone, they are less accurate. This is reflected in the quantitative values of the ADC which, correlated to PI-RADS, are less reliable in the transition zone than in the peripheral one.

To further investigate whether the PI-RADS ≥ 3 lesions’ ADC values can differentiate between clinically significant prostate cancer and benign lesions vs. not clinically significant cancer, we used ROC curve analysis (Figure 9). The AUC was 0.86 (0.85 for transition zone, 0.88 for peripheral zone) and suggested that the mean ADC value was a reasonable predictor for differentiating diagnoses of tumors with ISUP ≥ 2 and ISUP 1 or benign lesions (N) (Table 8).

Our study has some limitations; in fact, it cannot be excluded that errors occurred in the fusion procedure of the TRUS-MR images or, above all, during the biopsy, especially for smaller lesions or lesions located in regions of the gland that are difficult to access.

Fortunately, progress in artificial intelligence has opened new avenues for the diagnosis and management of prostate cancer. In particular, artificial intelligence technology can improve the performance and the precision of fusion biopsy [44].

In our study, the number of patients was relatively small, which may result in small differences in the ADC values for prostate tissues.

Moreover, the utilization of the freehand ROI for estimating the ADC values of smaller lesions may have been inaccurate.

Finally, our study did not evaluate the difference of ADC values in different MRI scanners. We used only one MRI scanner, so, we do not know how the ADC values would have read using different scanners.

## 5. Conclusions

In conclusion, our study confirms the growing significance of MRI for diagnosis and for the management of prostate cancer.

Our study, in particular, highlights the usefulness of the quantitative ADC in discriminating between not clinically significant (ISUP 1) and clinically significant prostate cancers (ISUP 2–5).

We also found a correlation between ADC values and PI-RADS groups.

Quantitative ADC derived from DWI sequences can be useful in the assessment of the aggressiveness of prostate cancer and could be an important tool in the evaluation of prostate MRI.

## Figures and Tables

**Table 1:** ISUP grades with respective Gleason scores.

ISUP Grade	Gleason Score
1	6 (3 + 3)
2	7 (3+ 4)
3	7 (4 + 3)
4	8 (4 + 4; 3+ 5; 5 + 3)
5	9 (4 + 5; 5 + 4) 10 (5 + 5)

**Table 2:** Mean ADC values ± standard deviation (SD) in benign (N) and in each ISUP group (from 1 to 5) lesions.

	Mean ADC Value	SD
N	1.00	±0.13
ISUP 1	0.79	±0.09
ISUP 2	0.76	±0.14
ISUP 3	0.73	±0.16
ISUP 4	0.72	±0.19
ISUP 5	0.72	±0.12

**Table 3:** Mean ADC ± SD in transition and peripheral zones in benign lesions (N), ISUP 1 lesions, and ISUP 2–5 lesions.

	N	ISUP 1	ISUP 2–5
Transition Zone	0.98 ± 0.12	0.74 ± 0.07	0.73 ± 0.14
Peripheral Zone	1.01 ± 0.14	0.84 ± 0.09	0.74 ± 0.15

**Table 4:** Spearman rank correlation (rho and *p*-value) between mean ADC values and ISUP groups in transition and peripheral zones.

	Spearman Rho	*p*-Value
Transition Zone	−0.67	1.23 × 10^−6^
Peripheral Zone	−0.64	1.77 × 10^−14^
Total	−0.65	3.92 × 10^−20^

**Table 5:** Mean ADC values ± SD in each PI-RADS groups from 3 to 5.

	Mean ADC Value	SD
PI-RADS 3	0.97	±0.13
PI-RADS 4	0.86	±0.18
PI-RADS 5	0.73	±0.18

**Table 6:** Mean ADC ± SD in transition and peripheral zones in each PI-RADS group (from 3 to 5).

	PI-RADS 3	PI-RADS 4	PI-RADS 5
Transition Zone	0.94 ± 0.15	0.87 ± 0.17	0.75 ± 0.16
Peripheral Zone	1.00 ± 0.11	0.85 ± 0.18	0.72 ± 0.18

**Table 7:** Spearman rank correlation (rho and *p*-value) between mean ADC values and PI-RADS groups in transition and peripheral zones.

	Spearman Rho	*p*-Value
Transition Zone	−0.31	0.04
Peripheral Zone	−0.50	2.07 × 10^−08^
Total	−0.47	1.03 × 10^−09^

**Table 8:** AUC value in each prostate zone.

	AUC
Transition Zone	0.85
Peripheral Zone	0.88
Total	0.86

## Data Availability

Interdisciplinary Department of Medicine, Section of Radiology and Radiation Oncology, University of Bari “Aldo Moro”, Bari, Italy.

## Abbreviations

MRI	Magnetic resonance imaging
ADC	Apparent diffusion coefficient
DWI	Diffusion-weighted imaging
ISUP	International Society of Urological Pathology
LUTS	Lowe urinary tract symptoms
BPH	Benign prostatic hypertrophy
RE	Rectal examination
PSA	Prostate-specific antigen
TRUS	Transrectal ultrasound
SNR	Signal-to-noise ratio
TURP	Transurethral resection of the prostate
DCE	Dynamic contrast enhanced
PACS	Picture Archiving and Communication System
ROI	Region of interest
SD	Standard deviation
ROC	Receiver operating characteristic
AUC	Area under the ROC curve
CT	Computed tomography
ACR	American College of Radiology
ESUR	European Society of Urogenital Radiology
